# Distribution of Carbapenemase Genes among Carbapenem-Non-Susceptible *Acinetobacter baumanii* Blood Isolates in Indonesia: A Multicenter Study

**DOI:** 10.3390/antibiotics11030366

**Published:** 2022-03-09

**Authors:** Dewi Anggraini, Dewi Santosaningsih, Yulia Rosa Saharman, Pepy Dwi Endraswari, Cahyarini Cahyarini, Leli Saptawati, Zinatul Hayati, Helmia Farida, Cherry Siregar, Munawaroh Pasaribu, Heriyannis Homenta, Enty Tjoa, Novira Jasmin, Rosantia Sarassari, Wahyu Setyarini, Usman Hadi, Kuntaman Kuntaman

**Affiliations:** 1 Doctoral Program, Faculty of Medicine, Universitas Airlangga, Surabaya 60115, Indonesia; dewi.anggraini-2019@fk.unair.ac.id; 2 Department of Microbiology, Faculty of Medicine, Universitas Riau, Pekanbaru 28133, Indonesia; novirajasminmd@gmail.com; 3 Arifin Achmad General Hospital, Pekanbaru 28156, Indonesia; 4 Department of Clinical Microbiology, Faculty of Medicine, Universitas Brawijaya, Malang 65145, Indonesia; dewi.santosa@ub.ac.id; 5 Dr. Saiful Anwar Hospital, Malang 65112, Indonesia; 6 Department of Clinical Microbiology, Faculty of Medicine, Universitas Indonesia, Jakarta 10320, Indonesia; yulia.rosa18@gmail.com; 7 Pelni Hospital, Jakarta 11410, Indonesia; 8 Department of Medical Microbiology, Faculty of Medicine, Universitas Airlangga, Surabaya 60115, Indonesia; pepy.dr@fk.unair.ac.id (P.D.E.); santisarassari@yahoo.com (R.S.); 9 Dr. Soetomo General Academic Hospital, Surabaya 60286, Indonesia; usmanhadi@sby.centrin.net.id; 10 Department of Clinical Microbiology, Persahabatan General Hospital, Jakarta 13230, Indonesia; ririn_cahya@yahoo.com; 11 Department of Microbiology, Faculty of Medicine, Universitas Sebelas Maret, Surakarta 57126, Indonesia; lelisaptawati@staff.uns.ac.id; 12 Department of Microbiology, Dr. Moewardi Teaching Hospital, Surakarta 57126, Indonesia; 13 Department of Microbiology, Faculty of Medicine, Universitas Syiah Kuala, Banda Aceh 23111, Indonesia; hayatikarmil@unsyiah.ac.id; 14 Department of Microbiology, Dr. Zainoel Abidin Hospital, Banda Aceh 24415, Indonesia; 15 Department of Microbiology, Faculty of Medicine, Diponegoro University, Semarang 50275, Indonesia; helmia.f@fk.undip.ac.id; 16 Dr. Kariadi Hospital, Semarang 50244, Indonesia; 17 H. Adam Malik Hospital, Medan 20136, Indonesia; drchrry@yahoo.com; 18 Ulin Hospital, Banjarmasin 70233, Indonesia; munawarohpasaribu@gmail.com; 19 Department of Clinical Microbiology, Faculty of Medicine, University of Sam Ratulangi, Manado 95115, Indonesia; herihomenta@unsrat.ac.id; 20 Department of Microbiology, School of Medicine and Health Sciences, Atma Jaya Catholic University of Indonesia, Jakarta 12930, Indonesia; enty@atmajaya.ac.id; 21 Institute of Tropical Disease, Universitas Airlangga, Surabaya 60115, Indonesia; wahyu_setyarini82@yahoo.com; 22 Department of Internal Medicine, Faculty of Medicine, Universitas Airlangga, Surabaya 60115, Indonesia

**Keywords:** infectious disease, *Acinetobacter baumannii*, CNSAB, carbapenemase gene, resistant factor, Indonesia

## Abstract

Carbapenem non-susceptible *Acinetobacter baumannii* (CNSAB) is an important pathogen that causes nosocomial bacteremia among critically ill patients worldwide. The magnitude of antibiotic resistance of *A. baumanii* in Indonesia is expected to be significant; however, the data available are limited. The aim of this study was to analyze the genetic profiles of CNSAB isolates from patients with bacteremia in Indonesia. CNSAB isolates from blood cultures of bacteremia patients in 12 hospitals in Indonesia were included. The blood cultures were conducted using the BacT/Alert or BACTEC automated system. The CNSAB were identified with either Vitek 2 system or Phoenix platform followed by a confirmation test using a multiplex polymerase chain reaction (PCR) assay, targeting the specific gyrB gene. The carbapenemase genes were detected by multiplex PCR. In total, 110 CNSAB isolates were collected and were mostly resistant to nearly all antibiotic classes. The majority of CNSAB isolates were susceptible to tigecycline and trimethoprim-sulfamethoxazole (TMP-SMX), 45.5% and 38.2%, respectively. The *bla*_OXA-51-like_ gene was identified in all CNSAB isolates. Out of the total, 83.6% of CNSAB isolates had *bla*_OXA-23-like_ gene, 37.3% *bla*_OXA-24-like_ gene, 4.5% *bla*_NDM-1_ gene, 0.9% *bla*_IMP-1_ gene, and 0.9% *bla*_VIM_ gene. No *bla*_OXA-48-like_ gene was identified. The *bla*_OXA-23-like_ gene was the predominant gene in all except two hospitals. The presence of the *bla*_OXA-24-like_ gene was associated with resistance to tigecycline, amikacin, TMP-SMX and cefoperazone-sulbactam, while *bla*_OXA-23-like_ gene was associated with resistance to TMP-SMX and cefoperazone-sulbactam. In conclusion, the *bla*_OXA-23-like_ gene was the predominant gene among CNSAB isolates throughout Indonesia. A continuous national surveillance system needs to be established to further monitor the genetic profiles of CNSAB in Indonesia.

## 1. Introduction

*Acinetobacter baumannii* (*A. baumannii*) is a non-motile, glucose-non-fermenting oxidase-negative Gram-negative bacterium [1]. It is recognized as a main pathogen causing hospital-acquired infections worldwide and is responsible for 2–10% of all Gram-negative hospital-acquired infections [2,3]. The infections are common in immunocompromised individuals, in particular those who have been hospitalized for a long time due to major surgical procedures or other underlying diseases [4,5]. The ability of this bacterium to thrive in a variety of environments with low nutrition is a key component of its pathogenesis [1]. *A. baumannii* can cause bacteremia, ventilator-associated pneumonia, infections of urinary tract, skin and soft tissues, burn and surgical wound infections, osteomyelitis and meningitis [1,6]. The bacteria could easily enter the human body through open wounds, intravascular catheters and mechanical ventilators [3,5]. The mortality rate of bacteremia due to *A. baumannii* ranges from 30–40% [7].

*A. baumannii* is intrinsically resistant and acquired resistant to various antibiotics [8]. It has a high level of intrinsic resistance to antimicrobial groups such as macrolides, lincosamides, glycopeptides and streptogramins [1]. In addition, it is also able to develop resistance to almost all classes of antibiotics. Some of the resistance mechanisms of *A. baumannii* include producing β-lactamases, overexpression of intrinsic antibiotic modifying enzymes, efflux pumps, permeability defects, and modifications of target sites of the antibiotics [1,9]. All these mechanisms lead to significant challenges in treating *A. baumannii* infections due to decreased number of available antibiotics in the clinical setting [1,9].

Carbapenems (imipenem, meropenem and doripenem) are considered as effective antibiotics for *A. baumannii* infection; however, resistance to this antibiotic class makes *A. baumannii*-associated infections difficult to be treated [10]. The prevalence of carbapenem non-susceptible *A. baumannii* (CNSAB) is increasing worldwide, including in Indonesia [11,12,13,14]. The main resistance mechanism of CNSAB is to produce β-lactamase enzymes [15,16]. β-lactamases that possess versatile hydrolytic capacities and therefore are able to hydrolyze carbapenems are called carbapenemases [15,16]. Some β-lactamases have carbapenemase activity and some do not. There are four major classes of β-lactamase based on molecular structure (class A, B, C and D) [16]. Class D β-lactamases, also known as oxacillinase (OXA), are the most common resistance factor against carbapenem in CNSAB isolates and have multiple subtypes [15,16]. OXA-51-like enzyme, that hydrolyzes carbapenems and penicillins, for example, is intrinsically possessed by *A. baumannii*. In addition, *A. baumannii* have acquired some carbapenemases including OXAs from the environment, and OXA-23-, OXA-40/24, and OXA-58-like enzymes are the most common detected in CNSAB. OXA-23-like enzyme is the most common driver of nosocomial outbreaks of CNSAB [15,16]. The less common causes of carbapenem resistance in CNSAB are class B β-lactamases such imipenemase (IMP), Verona integron-encoded metallo-β-lactamase (VIM), Seoul imipenemase (SIM) and New Delhi metallo-β-lactamase (NDM) [1,15,16]. 

Βeta-lactamases are encoded by β-lactamase genes (*bla*). Several studies have been conducted to assess the genetic profiles of CNSAB isolates around the globe. A study in France found that *bla*_OXA-23_ was the most dominant gene in isolated CNSAB (82%) [17]. In Saudi Arabia, a study found that *bla*_OXA-51_ was detected in all isolated CNSAB as an intrinsic gene and *bla*_OXA-23_ was found to be the most predominant gene [18]. The same findings were reported from China [19,20]. Meanwhile, a study found that the *bla*_OXA-23_ is not yet widespread among *A. baumannii* in Japan [21].

Indonesia has the fourth highest population globally, with thousands of islands across its archipelago. National surveillance on antimicrobial resistance is challenging to conduct in the country [22]. In addition, only limited data are available on the molecular epidemiology of CNSAB in Indonesia, and therefore the origin and spread of antimicrobial resistance patterns cannot be fully understood [11]. In this study, we determined the genetic profiles of CNSAB isolates associated with bacteremia patients in 12 hospitals across the Indonesian archipelago using multiplex polymerase chain reaction (PCR) assay.

## 2. Results

### 2.1. Distribution of CNSAB Isolates and Patients’ Characteristics 

**Figure 1:**
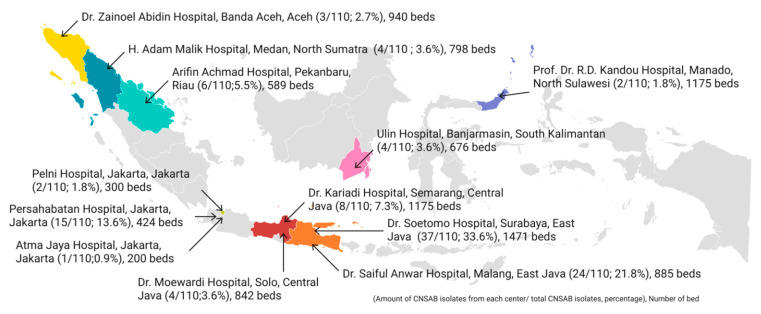
Distribution of CNSAB isolates with confirmed *gyrB* gene from each hospital in Indonesia (*n* = 110). The percentage is calculated as the number of *gyrB*-positive isolates from each hospital divided by the total number of isolates carrying the *gyrB* in this study. The number of beds from each hospital are provided to increase understanding of the profile of the study site.

A total of 123 CNSAB isolates were tested, of which 110 isolates carried the *A. baumannii*-specific *gyrB* gene, confirming the presence of *A. baumanii* species. The distribution of the CNSAB isolates based on the geographic locations and the number of beds of each hospital are provided in Figure 1.

The characteristics of the patients with confirmed CNSAB based on gender and age are provided in Table 1. More than half of the patients were male (60%, 66/110) and 62.7% of the patients were aged between 15–64 years old, with a mean age of 39.3 (±24.2) years. Most of the patients (76.3%) were treated at an intensive care unit (Table 1).

### 2.2. Antibiotic Resistance Test Results

**Figure 2:**
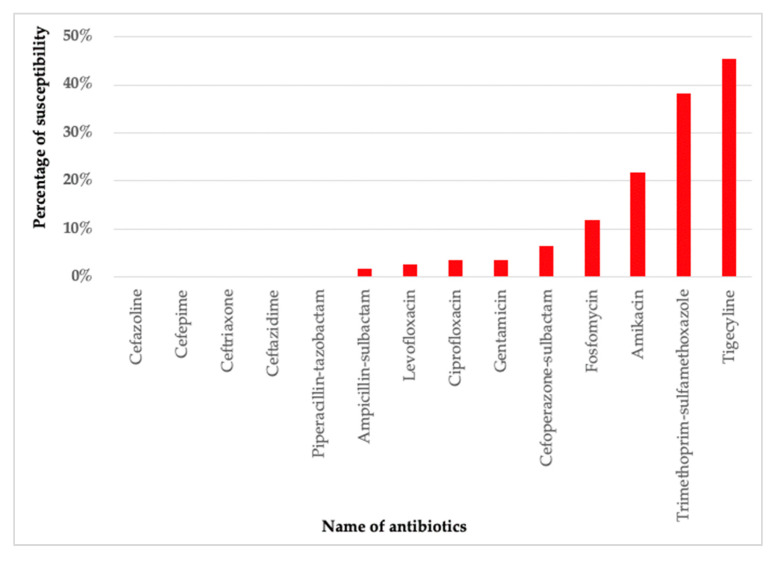
Antibiotic resistance test of CNSAB isolates from bacteremia patients in Indonesia (*n* = 110).

Our data suggested that tigecycline was the most susceptive to CNSAB isolates followed by trimethoprim-sulfamethoxazole (TMP-SMX), amikacin, and fosfomycin. Out of 110 CNSAB isolates, less than half were sensitive to tigecycline (45.5%, 50/110) and TMP-SMX (38.2%, 42/110) (Figure 2). Most of the CNSAB isolates were resistant to cephalosporins, fluroquinolone, aminoglycosides, and beta-lactam/beta-lactamase inhibitor antibiotics.

### 2.3. Distribution of the Carbapenemase Genes

This study detected seven carbapenemase genes which are commonly found in *A. baumannii*: *bla*_OXA-51-like_, *bla*_OXA-23-like_, *bla*_OXA-24-like_, *bla*_OXA-48-like_, *bla*_NDM-1_, *bla*_VIM_ and *bla*_IMP-1_, genes that encode for OXA-51-like, OXA-23-like, OXA-40/24-like, OXA-58-like, NDM-1, VIM, IMP-1 and IMP-1 enzyme. The *bla*_OXA-51-like_ gene, which is assumed to be an intrinsic gene of *A. baumannii* species, was detected in all CNSAB isolates (Table 2). The *bla*_OXA-23-like_ gene was the most prevalent carbapenemase gene detected (83.6%, 92/110), followed by *bla*_OXA-24-like_ gene (37.3%, 41/110). None of the isolates had the *bla*_OXA-48-like_ gene (Table 2). 

By excluding *bla*_OXA-51-like_, our data revealed that 24.5% of CNSAB isolates carried more than one carbapenemase gene (Table 2). There were 22.7% (25/110) that had two carbapenemase genes with four combinations: *bla*_OXA-23-like_/*bla*_OXA-24-like_ 21 isolates; *bla*_OXA-23-like_/*bla*_NDM-1_ two isolates; *bla*_OXA-24-like_/*bla*_NDM-1_ one isolate; and *bla*_OXA-23-like_/*bla*_IMP-1_ one isolate. Two (1.8%) isolates had three carbapenemase genes: *bla*_OXA-23-like_/*bla*_OXA-24-like_/*bla*_NDM-1_ and *bla*_OXA-23-like_/*bla*_OXA-24-like_/*bla*_VIM_ for each isolate (Table 2). 

**Figure 3:**
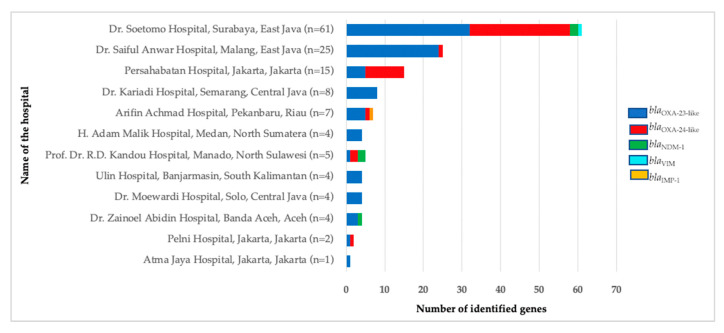
Carbapenemase genes’ distributions identified from 110 CNSAB isolates in 12 hospitals in Indonesia (*n* = 140). There are 140 genes identified from 110 isolated CNSAB. IMP: imipenemase, NDM: New Delhi metallo-beta-lactamase, OXA: oxacillinase, VIM: Verona integron-encoded metallo-beta-lactamase.

The *bla*_OXA-23-like_ gene was the most prevalent carbapenemase gene in all research centers except in Persahabatan Hospital in Jakarta and Kandou Hospital in North Sulawesi. In Persahabatan Hospital, *bla*_OXA-24-like_ gene was the most prevalent (66.7%, 10/15), while at Kandou Hospital, *bla*_OXA-24-like_ and *bla*_NDM-1_ were the most dominant genes (Figure 3).

When we divided the study sites into two categories: Java (populated and developed island) and outside Java (less-populated and less-developed islands), the *bla*_OXA-24-like_ gene was more dominant in Java compared to outside Java (41.8% vs. 15.8%), while *bla*_NDM-1_ and *bla*_IMP1-like_ were predominant outside Java (15.8% vs. 2.2% and 5.3% vs. 0.0%, respectively) (Table 3). 

### 2.4. Association between the Presence of Carbapenemase Genes and Antibiotic Resistance Tests 

Since the most frequent carbapenemase genes identified among the isolated were *bla*_OXA-23-like_ and *bla*_OXA-24-like_ genes, the associations between the presence of these genes with the pattern of antibiotic resistance results were assessed. Our data suggested that the presence of *bla*_OXA-23-like_ or *bla*_OXA-24-like_ had a significant association with susceptibility to some antibiotics (Table 4). The percentage of susceptibility to cefoperazone-sulbactam was significantly lower in *bla*_OXA-23-like_-positive isolates than *bla*_OXA-23-like_ negative isolates (1.1% vs. 33.3%, *p* < 0.001) However, in *bla*_OXA-23-like_-positive isolates, the percentage of susceptibility to TMP-SMX was significantly higher than CNSAB isolates without *bla*_OXA-23-like_ (44.6% vs. 5.6%; *p* < 0.001) (Table 4).

In CNSAB isolates that harbored *bla*_OXA-24-like_, the percentage of susceptibility to amikacin, tigecycline and TMP-SMX was significantly lower than the CNSAB isolates without *bla*_OXA-24-like_, by 7.3%% vs. 30.4%, 26.8% vs. 56.5%, and 17.1% vs. 50.7%, respectively. In contrast, the percentage of susceptible to cefoperazone-sulbactam was higher in *bla*_OXA-24-like_-positive isolates compared to *bla*_OXA-24-like_-negative isolates, at 14.6% vs. 1.4%; *p* = 0.010 (Table 4). Some antibiotic resistances were not associated with the presence of *bla*_OXA-23-like_ and *bla*_OXA-24-like_ genes (Table 4). 

## 3. Discussion

This is the first multicenter study assessing the molecular epidemiology of CNSAB in Indonesia. Our study found that 76.3% of the CNSAB were isolated from bacteremia patients treated in the intensive care unit (ICU). The risk of bacteremia due to CNSAB increases because patients in the ICU generally have low immunity, use invasive devices, have surgery or other underlying diseases, or receive broad-spectrum antibiotics [23]. In the present study, some patients were neonates treated in the neonatal intensive care unit (NICU). Studies have reported that bacteremia-associated CNSAB were predominantly from those treated in the ICU and NICU [24,25,26,27,28].

Our study found that the CNSAB isolates were resistant to almost all classes of antibiotics such as cephalosporins, fluroquinolones, beta-lactam/beta-lactamase inhibitors and aminoglycosides. Our data found that tigecycline had the best sensitivity (45.45%). This antibiotic is used extensively in treating CNSAB-associated infections, usually in combination with other antibiotics. However, the efficacy of this antibiotic is still debated because it is bacteriostatic and has poor pharmacokinetics in the bloodstream and lungs [29,30], and therefore, in general, tigecycline is not recommended for bacteremia [30]. The sensitivity of tigecycline in our study was lower than in another study [31], possibly because tigecycline has been widely used in Indonesia.

The second highest sensitivity for CNSABs was TMP-SMX (38.18%). This sensitivity rate is relatively high in comparison with a previous review reporting that 22 out of 26 studies found that the CNSAB resistance rate to TMP-SMX was more than 80% [32]. This difference may be because TMP-SMX is only available in oral form in Indonesia, and the use of this antibiotic is therefore limited in outpatient settings to treat gastroenteritis or non-complicated urinary tract infections. Although antibiotic prescription and use in the outpatient setting is problematic, there is a need to measure and improve how clinicians prescribe and patients use antibiotics [33]. This data suggests that TMP-SMX is still a promising therapeutic option for CNSABs in Indonesia. A study found that TMP-SMX treatment in patients with severe infection was associated with a better clinical improvement and shorter hospital stay compared to colistin or ampicillin-sulbactam [34].

Other antibiotic options for CNSABs such as colistin and minocycline are not commercially available in Indonesia. The sensitivity tests for these two antibiotics are not routinely conducted in the country and therefore no sensitivity data are available for these two antibiotics. The high level of CNSAB resistance to all tested antibiotics in this study urges that colistin and minocycline, or a new class of antibiotic, are urgently needed to treat CNSAB infection in Indonesia.

In our study, besides the intrinsic *bla*_OXA-51-like_ gene, the most prevalent carbapenemase gene identified in CNSAB isolates was *bla*_OXA-23-like_. This is consistent with previous small studies from Indonesia [24,35] and other countries around the world [31,36,37,38,39]. Overall, 24.6% of CNSAB isolates had more than one carbapenemase gene, and this could contribute to an increase in minimum inhibitory concentration (MIC) due to higher hydrolytic activity, which may eventually lead to high resistance to beta lactam antibiotics [38]. The *bla*_OXA-24-like_ gene was the second most prevalent in our study, and this gene was predominant in two hospitals: Persahabatan Hospital and Kandou Hospital, located in Jakarta and North Sulawesi, respectively. This is not surprising, since studies in other countries such as Spain and Portugal also have found that *bla*_OXA-24-like_ could be the predominant gene [40,41]. 

Based on geographical location, our study suggested that the distribution of *bla*_OXA-24-like_ and *bla*_NDM-1_ genes are different between Java and outside Java. Java, where the capital city Jakarta is located, has the largest population density and population mobility in Indonesia. The health services and facilities in Java are also more comprehensive than outside Java. Further investigation is warranted to determine the disproportional distribution of the carbapenemase genes between these two regions. 

Several studies have been conducted to investigate the relationship between genotype profiles and drug resistance of multidrug-resistant *A. baumannii*. A study conducted in Vietnam found that, with the exception of TMP-SMX, resistance to most antimicrobial agents was significantly related to the presence of *bla*_OXA-23_ gene [42]. A study in Saudi Arabia found there was an association between resistance to gentamicin and amikacin with the acquisition of insertion sequence *ISAba1* upstream of *bla*_OXA-51-like_ gene [31]. Insertion sequence *ISAba1* is associated with overexpression of the carbapenem genes [42]. Another study found that *A. baumannii* strains with two or more carbapenem genes had a high chance of being multidrug resistant [42]. Our study found that the presence of the carbapenem genes were associated with resistance to some antibiotics (Table 4). For example, the presence of *bla*_OXA-24-like_ was associated with resistance to amikacin, tigecycline and TMP-SMX, while *bla*_OXA-23-like_ was associated with resistance to cefoperazone-sulbactam. OXA-24-like enzyme has the ability to hydrolyze penicillin and has weaker activity against cephalosporins and carbapenems [1]. A study found that the *bla*_OXA-24–like_ gene was almost completely resistant to amikacin, cefepime, ceftazidime, piperacillin/tazobactam and ampicillin/sulbactam [43]. Meanwhile, OXA-23-like enzyme has the ability to hydrolyze carbapenems more significantly than other class D carbapenemase enzymes. This enzyme also can hydrolyze oxyimino cephalosporins, aztreonam, oxacillin, piperacillin and aminopenicillins [1]. The mechanism through which OXA-23- and OXA-24-like enzyme impact tigecycline and TMP-SMX resistance is currently unclear. 

This study has some limitations that need to be explained. Each study site had an uneven number of CNSAB isolates, which might distort the percentage of genes in each hospital. This is probably because the hospitals are different sizes. Second, due to unavailability of the antibiotics in the country, the current study is unable to provide sensitivity tests for colistin and minocycline against CNSAB.

## 4. Materials and Methods

### 4.1. Study Setting 

A multicenter cross-sectional study was conducted in 12 hospitals in nine provinces across the Indonesian archipelago between September 2019 and March 2021. Out of the total hospitals, 10 of them serve as provincial referral hospitals. The study covered four Indonesian main islands: seven hospitals from Java, three hospitals from Sumatra, and one hospital each from Borneo (Kalimantan) and Sulawesi. 

### 4.2. Bacterial Isolates

All CNSAB isolates were isolated from blood cultures of bacteremia patients. If more than one isolate was isolated from a patient, only the first isolate was analyzed in this study. Blood cultures were performed using the BacT/Alert (bioMérieux, Marcy l’Etoile, France) or BACTEC automated system (Becton Dickinson, Franklin Lakes, NJ, USA). The positive results were sub-cultured onto blood and MacConkey agar for further identification and antibiotic resistance tests, as recommended previously [24]. Identification and antibiotic resistance tests were carried out using an automated Vitek 2 System (bioMérieux, Marcy l’Etoile, France) or BD Phoenix (BD, Franklin Lakes, NJ, USA), followed the manufacturers’ protocols. The tested antibiotics were ampicillin-sulbactam, piperacillin-tazobactam, cefazoline, ceftriaxone, ceftazidime, cefepime, cefoperazone-sulbactam, levofloxacin, ciprofloxacin, fosfomycin, gentamicin, amikacin, tigecycline, and TMP-SMX. The interpretation for tigecycline was referred to the FDA, while interpretation for cefoperazone-sulbactam was based on the package insert breakpoints [44]. The antibiotics that were not included in those automated platforms were tested using the Kirby Bauer disc diffusion method [45], in which cefoperazone-sulbactam and fosfomycin discs were 105 μg and 200 μg, respectively. The interpretation followed the Clinical and Laboratory Standards Institute (CLSI) [46]. CNSAB was defined if the culture had intermediate or resistance to one of the carbapenems (meropenem, imipenem or doripenem). All laboratories participating in this study included the quality control strains: *Escherichia coli* ATCC 25,922 and *Pseudomonas aeruginosa* ATCC 27853.

### 4.3. Confirmatory Test for A. baumannii 

The molecular test to confirm the presence *A. baumannii* species was conducted at the Institute of Tropical Disease (ITD) at Universitas Airlangga in Surabaya. DNA extraction was carried out using the boiling method [47]. A multiplex PCR assay targeting *A. baumannii*-specific *gyrB* gene was used to confirm the *A. baumannii* species genotypically, as previously described [48,49]. The method is robust, reproducible, and cheaper than sequencing, and it enables us to identify most clinically relevant *Acinetobacter* species [48,49]. Two pairs of primers, designed to identify the *gyrB* gene of *A. baumannii* species [48,49], were used (Table 5).

The PCR amplification was carried out in 20 µL reaction solution consisting of 10 µL PCR mixture GoTaq Master Mix (Promega, Madison, WI, USA), 10 pM of each primer, 3 µL H_2_O, and 5 µL of DNA template. Amplification was performed with an initial denaturation at 94 °C for 2 min, followed by 25 cycles consisting of denaturation at 94 °C for 1 min, annealing at 60 °C for 30 s, and extension at 72 °C for 1 min, with a final extension at 72 °C for 10 min [48]. 

### 4.4. Detection of Carbapenemase Genes

CNSAB isolates containing the *gyrB* gene were tested to detect the *bla*_OXA-51-like_ gene and six other carbapenemase genes, which were divided into two multiplex PCR groups: one for detecting class B carbapenemases (*bla*_IMP-1_, *bla*_VIM_ and *bla*_NDM-1_) and one for class D carbapenemases (*bla*_OXA-23-like_, *bla*_OXA-24-like_ and *bla*_OXA-48-like_) [55]. The IMP and NDM have several sub-types. However, only IMP-1 and NDM-1 were assessed in this study because both are the main enzymes from IMP and NDM group in *A. baumannii*. The *bla*_OXA-23-like_, *bla*_OXA-24-like_ and *bla*_OXA-48-like_ genes were included since they are commonly reported from other countries including countries in Southeast Asia [15,16,17,18,19,20].

The primers to detect the carbapenemase genes are listed in Table 5. The amplification of all genes used a GoTaq PCR Master Mix (Promega, Madison, WI, USA). For *bla*_OXA-51_ gene detection, the total reaction volume was 25 µL, consisting of 12.5 PCR Master Mix, 10 pM forward and reverse primer, 5.5 H_2_O and 5 µL DNA template. The amplification had 33 cycles with denaturation at 94 °C for 55 s, annealing at 46 °C for 45 s, and extension at 72 °C for 60 s [36]. For amplification of class B carbapenemase genes, the total reaction volume was 20 µL, consisting of 12.5 PCR Master Mix, 10 pM of each primer, 0.7 µL H_2_O and 5 µL DNA template. The amplification conditions consisted of initial denaturation at 95 °C for 5 min, followed by 30 cycles of denaturation at 95 °C for 30 s, annealing at 58 °C for 30 s and extension at 72 °C for 30 s. The composition of the PCR mixture to detect class D carbapenemase genes was similar to class B. However, the amplification had slightly different conditions, with denaturation at 94 °C for 25 s, annealing at 52 °C for 40 s, and extension at 72 °C for 25 s. The amplicons of PCR amplifications were electrophoresed through ethidium bromide-stained 2% agarose gels at a voltage of 100 volts for 30 min. Visualization was conducted using a UV transilluminator and documented using a digital camera. Each PCR assay was accompanied with positive and negative controls. The positive control was obtained from the laboratory collection of the Institute of Tropical Disease at Universitas Airlangga. 

### 4.5. Statistical Analysis

The association between the presence of carbapenemase genes and the antibiotic resistance profile was analyzed with chi-squared test. A *p*-value less than 0.05 was considered as significant. All analyses were conducted using a Statistical Package for the Social Sciences software version 28 (SPSS for Windows, Chicago, IL, USA).

## 5. Conclusions

The *bla*_OXA-23-like_ gene is the most predominant gene in CNSAB isolates throughout Indonesia. The antibiotic resistance profiles of CNSAB in Indonesia are very worrying, and the distribution of the carbapenemase genes should receive more attention in the country. Continuous surveillance of antibiotic resistance in Indonesia needs to be strengthened to provide data on the antibiotic resistance burden in order to formulate a policy in the country and in the regions. Optimizing policy on the use of antibiotics and infection prevention and control in hospitals is very important and should be be implemented throughout the country.

## Figures and Tables

**Table 1:** Characteristics of the bacteremia patients with confirmed CNSAB (*n* = 110).

Characteristics	Group	Frequency (%)
Sex	Male	66 (60.0)
	Female	44 (40.0)
Age (years)	<1	17 (15.5)
	1–14	10 (9.1)
	15–64	69 (62.7)
	65–70	10 (9.1)
	>70	4 (3.6)
Unit	Intensive care unit	84 (76.3)
	Non-intensive care unit	26 (24.7)

**Table 2:** Distribution of the carbapenemase genes in CNSAB isolates from bacteremia patients in Indonesia (*n* = 110).

Gene Distribution	Frequency (%)
*bla*_OXA-51-like_	110 (100.0)
*bla*_OXA-23-like_	92 (83.6)
*bla*_OXA-24-like_	41 (37.3)
*bla*_OXA-48-like_	0 (0.0)
*bla*_NDM-1_	5 (4.5)
*bla*_VIM_	1 (0.9)
*bla*_IMP-1_	1 (0.9)
**Gene combinations**	
One gene	83 (75.5)
*bla*_OXA-23-like_	66 (60.9)
*bla*_OXA-24-like_	17 (15.5)
**Two-gene combinations**	**25 (22.7)**
*bla*_OXA-23-like_/*bla*_OXA-24-like_	21 (19.1)
*bla*_OXA-23-like_/*bla*_NDM-1_	2 (1.8)
*bla*_OXA-24-like_/*bla*_NDM-1_	1 (0.9)
*bla*_OXA-23-like_/*bla*_IMP-1_	1 (0.9)
**Three-gene combinations**	**2 (1.8)**
*bla*_OXA-23-like_/*bla*_OXA-24-like_/*bla*_NDM-1_	1 (0.9)
*bla*_OXA-23-like_/*bla*_OXA-24-like_/*bla*_VIM_	1 (0.9)

IMP: imipenemase, NDM: New Delhi metallo-beta-lactamase, OXA: oxacillinase, VIM: Verona integron-encoded metallo-beta-lactamase.

**Table 3:** Distribution of carbapenemase genes in CNSAB isolates based on geographics (Java vs. outside Java).

Carbapenemase Gene	Java (*n* = 116)	Outside Java (*n* = 24)
	Frequency (%)	Frequency (%)
*bla*_OXA-23-like_	75 (82.4)	17 (89.5)
*bla*_OXA-24-like_	38 (41.8)	3 (15.8)
*bla*_NDM-1_	2 (2.2)	3 (15.8)
*bla*_VIM_	1 (1.1)	0 (0.0)
*bla*_IMP-1_	0 (0.0)	1 (5.3)

IMP: imipenemase, NDM: New Delhi metallo-beta-lactamase, OXA: oxacillinase, VIM: Verona integron-encoded metallo-beta-lactamase.

**Table 4:** Association between the presence of *bla*_OXA-23-like_ and *_bla_*_OXA-24-like_ genes with antibiotic sensitivity tests.

Name of Antibiotic	Antibiotic Susceptibility Result	*bla*_OXA-23-like_		*p*-Value	*bla*_OXA-24-like_		*p*-Value
		Detected	Not Detected		Detected	Not Detected	
Ampicillin-sulbactam	Susceptible	2 (2.2)	0 (0.0)	1.000	0 (0.0)	2 (2.9)	0.528
	Non-susceptible	90 (97.8)	18 (100)		41 (100)	67 (97.1)	
Piperacillin-tazobactam	Susceptible	0 (0.0)	0 (0.0)	NA	0 (0.0)	0 (0.0)	NA
	Non-susceptible	92 (100)	18 (100)		41 (100)	68 (100)	
Cefazoline	Susceptible	0 (0.0)	0 (0.0)	NA	0 (0.0)	0 (0.0)	NA
	Non-susceptible	92 (100)	18 (100)		41 (100)	69 (100)	
Ceftriaxone	Susceptible	0 (0.0)	0 (0.0)	NA	0 (0.0)	0 (0.0)	NA
	Non-susceptible	92 (100)	18 (100)		41 (100)	69 (100)	
Ceftazidime	Susceptible	0 (0.0)	0 (0.0)	NA	0 (0.0)	0 (0.0)	NA
	Non-susceptible	92 (100)	18 (100)		41 (100)	69 (100)	
Cefepime	Susceptible	0 (0.0)	0 (0.0)	NA	0 (0.0)	0 (0.0)	NA
	Non-susceptible	92 (100)	18 (100)		41 (100)	69 (100)	
Gentamicin	Susceptible	3 (3.3)	1 (5.6)	0.516	1 (2.4)	3 (4.3)	1.000
	Non-susceptible	89 (96.7)	17 (94.4)		40 (97.6)	66 (95.7)	
Amikacin	Susceptible	22 (23.9)	2 (11.1)	0.352	3 (7.3)	21 (30.4)	0.005 *
	Non-susceptible	70 (76.1)	16 (88.9)		38 (92.7)	48 (69.6)	
Ciprofloxacin	Susceptible	4 (4.3)	0 (0.0)	1.000	0 (0.0)	4 (5.8)	0.295
	Non-susceptible	88 (95.7)	18 (100)		41 (100)	65 (94.2)	
Levofloxacin	Susceptible	3 (3.3)	0 (0)	1.000	0 (0)	3 (4.3)	0.292
	Non-susceptible	89 (96.7)	18 (100)		41 (100)	66 (95.7)	
Tigecycline	Susceptible	41(44.6)	9 (50.0)	0.672	11 (26.8)	39 (56.5)	0.002 *
	Non-susceptible	51 (55.4)	9 (50.0)		30 (73.2)	30 (43.5)	
Trimethoprim-sulfamethoxazole	Susceptible	41 (44.6)	1 (5.6)	0.002 *	7 (17.1)	35 (50.7)	<0.001 **
	Non-susceptible	51 (55.4)	17 (94.4)		34 (82.9)	34 (49.3)	
Fosfomycin	Susceptible	11 (12.0)	2 (11.1)	1.000	6 (14.6)	7 (10.1)	0.547
	Non-susceptible	81 (88.0)	16 (88.9)		35 (85.4)	62 (89.9)	
Cefoperazone-sulbactam	Susceptible	1 (1.1)	6 (33.3)	<0.001 **	6 (14.6)	1 (1.4)	0.010 *
	Non-susceptible	91 (98.9)	12 (66.7)		35 (85.4)	68 (98.6)	

* Significant at *p* < 0.05; ** Significant at *p* < 0.001; NA: not applicable, OXA: oxacillinase.

**Table 5:** Primers used to identify the *Acinetobacter baumannii* species and carbapenemase genes.

Target Gene	Primer Sequence (5′–3′)	Amplicon (Base Pair)	Reference
*gyrB*	Sp2F: GTTCCTGATCCGAAATTCTCG	490	[48,49]
	Sp4R: AACGGAGCTTGTCAGGGTTA		
	Sp4F: CACGCCGTAAGAGTGCATTA	294	[48,49]
	Sp4R: AACGGAGCTTGTCAGGGTTA		
*bla*_OXA-51-like_	F: ATGAACATTAAAGCACTCTTAC	825	[36]
	R: CTATAAAATACCTAATTGTTCT		
*bla*_IMP-1_	F: CTACCGCAGCAGAGTCTTTG	587	[50]
	R: AACCAGTTTTGCCTTACCAT		
*bla*_VIM_	F: TGGGCCATTCAGCCAGAT C	510	[50,51]
	R: ATGGTGTTTGGTCGCATATC		
*bla*_NDM-1_	F: CTGAGCACCGCATTAGCC	754	[52]
	R: GGGCCGTATGAGTGATTGC		
*bla*_OXA-23-like_	F: GATCGGATTGGAGAACCA GA	501	[53]
	R: ATTCTTGACCGCATTTCCAT		
*bla*_OXA-24-like_	F: GGTTAGTTGGCCCCCTTAAA	246	[53]
	R: AGTTGAGCGAAAAGG GGATT		
*bla*_OXA-48-like_	F: TTGGTGGCATCGATTATCGG	744	[54]
	R: GAGCACTTCTTTTGTGATGGC		
	R: CCCCTCTGCGCTCTACATAC		

F: forward primer; R: reverse primer.

## Data Availability

The underlying data of this study are available from the corresponding author on request.
